# Proton Therapy in the Treatment of Men with Breast Cancer

**DOI:** 10.14338/IJPT-23-00007.1

**Published:** 2023-10-25

**Authors:** Julie A. Bradley, Jayden Gracie, Raymond B. Mailhot Vega, Eric D. Brooks, Tenna Burchianti, Oluwadamilola T. Oladeru, Xiaoying Liang, Nancy P. Mendenhall

**Affiliations:** 1 Department of Radiation Oncology, University of Florida College of Medicine, Jacksonville, FL, USA; 2 Department of Radiation Oncology, Vanderbilt University Medical Center, Nashville, TN, USA; 3 University of Florida Health Proton Therapy Institute, Jacksonville, FL, USA; 4 Department of Radiation Oncology, Mayo Clinic, Jacksonville, FL, USA

**Keywords:** male breast cancer, proton therapy, radiation therapy, cardiac toxicity

## Abstract

**Purpose:**

Male breast cancer treatment involves multimodality therapy, including radiation therapy; nevertheless, few men have received proton therapy (PT) for it. Further, heart disease is an established leading cause of death in men, and radiation therapy heart dose correlates with cardiac toxicity, highlighting the need for cardiac-sparing radiation techniques. Thus, we provide a descriptive analysis of PT in a male breast cancer cohort.

**Patients and Methods:**

Men who received PT for localized breast cancer between 2012 and 2022 were identified from a prospective database. Toxicities were prospectively recorded by using the Common Terminology Criteria for Adverse Events (CTCAE), version 4.0.

**Results:**

Five male patients were identified. All had estrogen receptor (ER)–positive, Her2neu-negative disease and received adjuvant endocrine therapy. One had genetic testing positive for *BRCA2*, one had a variant of unknown significance (VUS) in the *APC* gene, and one had a VUS in *MSH2*. Median age was 73 years (range, 41–80). Baseline comorbidities included obesity (n = 1), diabetes (n = 1), hypertension (n = 4), history of deep vein thrombosis (n = 1), personal history of myocardial infarction (n = 3; 1 with a pacemaker), and a history of lung cancer (n = 1). All received PT to the left chest wall and comprehensive regional lymphatics. One received passive-scattering PT, and 4 received pencil beam scanning. One patient received a boost to the mastectomy incision via electrons. Median heart dose was 1 GyRBE (range, 0–1.0), median 0.1-cm^3^ dose to the left anterior descending artery was 7.5 GyRBE (range, 0–14.2), and median follow-up was 2 years (range, 0.75–6.5); no patient experienced a new cardiac event, and all remain free from breast cancer recurrence and progression.

**Conclusion:**

In a small case series for a rare diagnosis, PT to the chest wall and regional lymphatics, including internal mammary nodes, resulted in low cardiac exposure, high local regional disease control rates, and minimal toxicity. Proton therapy should be considered for treating men with breast cancer to achieve cardiac sparing.

## Introduction

With breast cancer constituting the most common noncutaneous cancer in women worldwide, the disease is often misconstrued as one that only affects females, thus excluding a specific focus on men with breast cancer. While most clinical studies analyze outcomes of women with breast cancer, it is assumed that men receive similar benefits from technologic advancements in radiation therapy, as many commonalities exist between male and female thoracic anatomy and breast cancer [1, 2].

With 2650 cases of breast cancer diagnosed in males compared to 281 550 cases in females in 2021 [3], breast cancer in males is relatively rare. While comprising only 1% of all breast cancers, the incidence of men with breast cancer is rising [4, 5]. Breast cancer treatment for men follows the same treatment approach as for women: often surgery followed by chemotherapy, endocrine therapy, or radiation therapy as indicated by disease characteristics, with neoadjuvant systemic therapy recommended for cases of advanced disease [2, 6]. Yadav et al [7] examined treatment patterns and prognostic factors for 10 873 men with breast cancer between 2004 and 2014, using the National Cancer Data Base, and found that 71.3% were treated with total mastectomies versus 23.7% with breast-conserving surgery; overall, 39.4% received adjuvant radiation therapy, 62.3% received hormone therapy, and 44.5% received chemotherapy. However, only 0.1% of men (n = 13) received proton therapy.

The breast tissue in males is less dense than in females, but the factors affecting the malignant changes are similar [6]. While there may be some biologic differences in breast cancer in men compared to women [8], the challenge in radiation therapy regarding optimal target coverage while minimizing heart dose exists for both sexes. Proton therapy provides a potential solution for minimizing cardiac exposure, particularly in people with left-sided breast cancer [9]. Minimizing cardiac toxicity is critical, as heart disease is the cause of death for 1 in 4 males in the United States [10]. The death rate from heart disease is higher for males in the United States than females. While this rate decreased for both sexes among persons aged 45 to 64 years from 1999–2011, it increased 3% for males and 7% for females from 2011–17, with a cardiac death rate of 189.8 per 100 000 males and 80.1 per 100 000 females in 2017 [11]. Most data on treatment for males with breast cancer are extrapolated from studies that solely or predominantly included females [6]. Reports on the use of proton therapy for breast cancer in men are limited. This case series describes men with left-sided breast cancer treated with proton therapy.

## Materials and Methods

This prospective outcome study was performed under an institutional review board–approved protocol at the University of Florida Health Proton Therapy Institute. Patients included in this analysis were males treated with proton therapy between 2012 and 2022.

Contouring and treatment planning were completed by using MIM (MIM Software, Cleveland, Ohio), Eclipse (Varian Medical Systems, Palo Alto, California), and RayStation (RaySearch Laboratories, Stockholm, Sweden) software. Target contouring was conducted in alignment with the RADCOMP Atlas [12]. The goal target coverages for the chest wall and each of the nodal clinical target volumes were 95% of the dose to 95% of the volume. Plan robustness was performed per institutional protocol. For patients who received passive-scattering proton therapy, a beam-specific planning target volume (BsPTV) was generated to account for setup and range uncertainty. The expansion of the BsPTV was 5 mm in the direction perpendicular to the beam angle, with a 2.5% range + 1.5 mm in the proximal and distal directions. For those who received proton pencil beam scanning (PBS) therapy, robust optimization with 5-mm setup uncertainty and 3.5% range uncertainty was used. Robustness evaluation was performed for both passive-scattering and PBS plans to ensure plan robustness.

Patient, disease, and treatment data were reviewed and collected by using electronic medical records, imaging, and treatment planning dosimetry to determine patient, tumor, and treatment characteristics. Toxicity data were prospectively recorded by using the Common Terminology Criteria for Adverse Events (CTCAE), version 4.0 (US National Cancer Institute, Bethesda, Maryland), and obtained in retrospective chart review from the electronic medical record.

Photon plans were designed for dosimetric comparison for 4 patients. One patient received photon therapy for a portion of his treatment course. The same institutional planning guidelines were used to direct proton and photon planning.

## Results

Five male patients were identified, ranging in age from 41 to 80 years. All had estrogen receptor (ER)–positive, Her2neu-negative disease and received adjuvant endocrine therapy. One patient had genetic testing positive for a pathogenic mutation in *PALB2*. Baseline comorbidities included obesity (n = 1), diabetes (n = 1), hypertension (n = 4), history of deep vein thrombosis (DVT) (n = 1), personal history of myocardial infarction (n = 3; 1 with a pacemaker), and history of lung cancer (n = 1).

**Figure 1.**
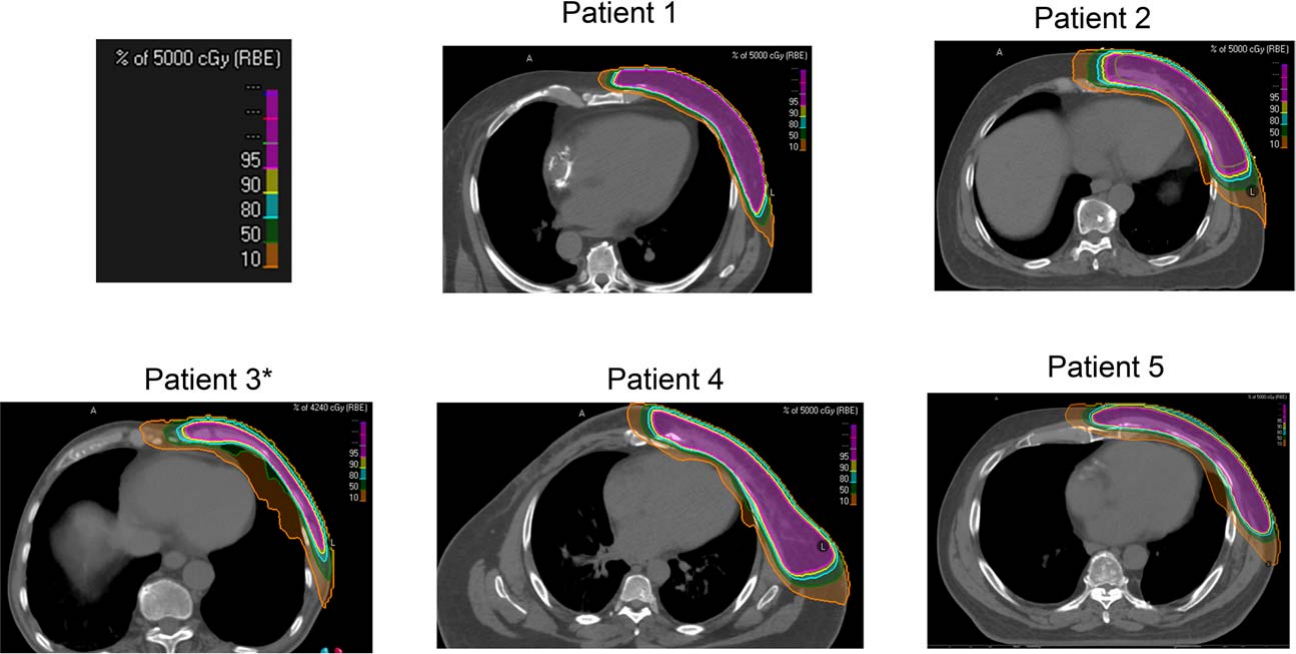
The radiation proton dosimetry for each patient on a representative axial CT slice, shown in colorwash by percentage isodose line for the initial phase (prescription dose of 50 Gy for patients 1, 2, 4, and 5; and 42.4 Gy for patient 3): 95%, pink; 90%, yellow; 80%, light blue; 50%, green; 10%, orange. *This patient’s plan shows the percentage isodose lines for a prescription dose of 42.4 Gy. Abbrevations: CT, computed tomography; Gy, Gray.

**Figure 2.**
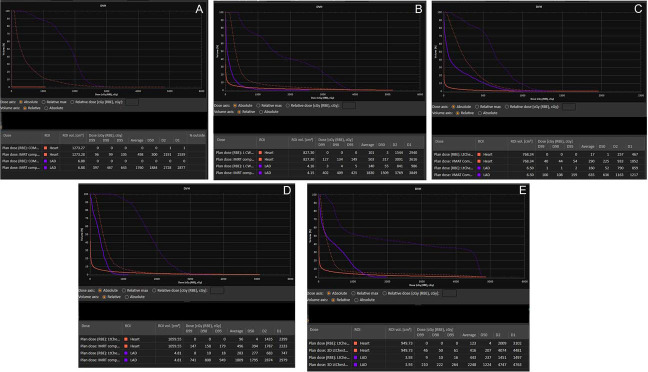
DVH for whole heart (orange) and LAD (purple) for proton (solid line) versus photon (dashed line) plans for patients 1 to 5 (A-E, respectively). Abbreviations: DVH, dose-volume histogram; LAD, left anterior descending artery.

**Figure 3.**
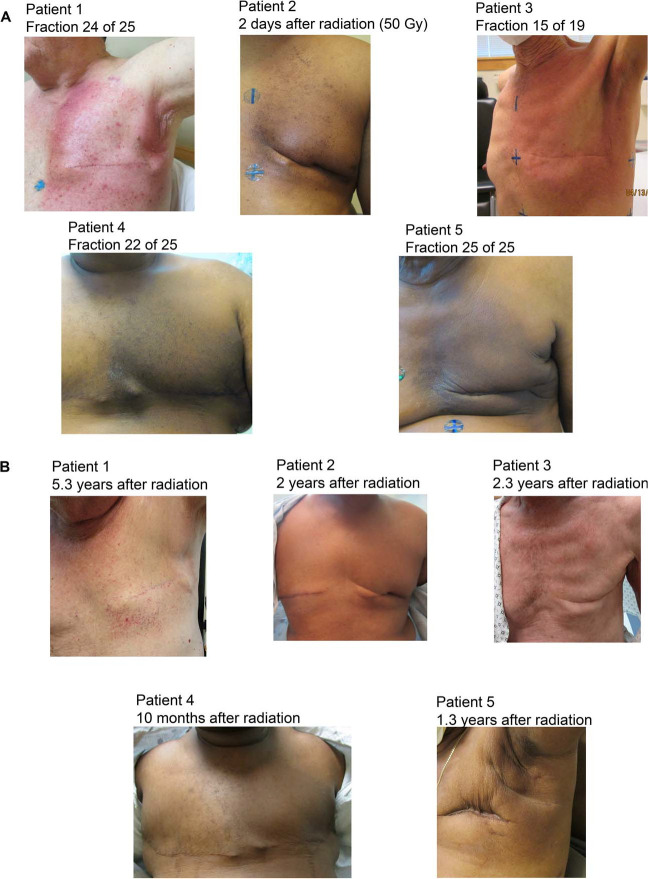
Clinical photographs of the chest near the end of radiation therapy (A) and post radiation (B).

The details of each case are described below and summarized in **Tables 1** and **2**. All patients received proton therapy (passive-scattering, n = 1; PBS, n = 4) to the left chest wall and comprehensive regional lymphatics (including internal mammary nodes, supraclavicular nodes, and axilla levels I, II, and III). One patient received a component of photon therapy (9 fractions of three-dimensional [3D] conformal photons using breath-holding techniques) while insurance coverage for proton therapy was undergoing authorization. All patients were treated in the supine position. No patients were treated with breath-hold or gating for proton delivery. **Figure 1** demonstrates the radiation dosimetry, **Figure 2** depicts the cardiac dose-volume histogram for each patient, and **Figure 3** provides clinical photographs of the treated area at the end of radiation and last follow-up.

**Table 1.** Patient characteristics, treatment, and outcomes (N = 5).

Characteristic	Patient				
	1	2	3	4	5
Age, y	75	60	80	41	73
Comorbidities	DM, OSA, myasthenia gravis, hypertriglyceridemia, hypertension, CAD, prior MI, atrial fibrillation, pacemaker	Prior MI, history of bacterial meningitis, depression, DVT, hyperlipidemia, hypertension	None	Obesity, hypertension, hypercholesterolemia, OSA	Hypertension, prior MI, prior lung cancer
Genetic testing	Negative	VUS in *APC* gene	Negative	PALB2+; VUS in *CDH1*	VUS in *MSH2* gene
Clinical stage	T2 N0 M0	Left: T2 N0 M0; right: Tis N0 M0	T2 N1 M0	T2 N0 M0	Initial: T1c N0 M0; recurrent: Tx N1 M0
Pathologic stage	T2 N1a	Left: pT2 N1mic; right: pTis N0	T1c N3a	T2 N0 M0	Initial: T1b Nx; recurrent: T0 N1a
Breast cancer laterality	Left	Bilateral	Left	Left	Left
Tumor location	Retroareolar	Retroareolar	Retroareolar	Retroareolar	Retroareolar
Histology	Grade 2 IDC	Grade 1 IDC (left)	Grade 2 IDC	Grade 3 IDC	Grade 3 IDC
Receptor status	ER-positive (100%); PR-positive (65%); HER2-negative; Ki-67, 10%	ER-positive (90%); PR-positive (50%); HER2-negative	ER-positive (>50%); PR-positive (>50%); HER2-negative; Ki-67 > 20%	ER-positive (>90%); PR-negative; HER2-negative; Ki-67 > 20%	ER-positive (>89%); PR-negative; HER2-negative
Treatment course	Left mastectomy + SLNB → RT → letrozole	Bilateral mastectomy + SLNB → chemo (docetaxel × 4) → RT → tamoxifen	Tamoxifen -> left MRM -> chemo (TC) → RT → anastrozole → letrozole → capecitabine (ongoing)	Left mastectomy + SLNB → RT → tamoxifen	Left mastectomy + SLNB (no nodes obtained) → tamoxifen → axillary recurrence treated with ALND → chemo with docetaxel × 4 → RT → letrozole + leuprorelin
Follow-up, y	6.5	2	2.8	0.8	1.5
Late toxicity	Grade 1 telangiectasia	Lymphedema, resolved with therapies	Lymphedema (preceded RT)	None	Mild chest wall pain, decreased ROM, lymphedema (preceded RT)
Status	NED	NED	NED	NED	NED

**Abbreviations:** DM, diabetes mellitus; OSA, obstructive sleep apnea; CAD, coronary artery disease; MI, myocardial infarction; DVT, deep vein thrombosis; VUS, variant of uncertain significance; APC, adenomatous polyposis coli; IDC, invasive ductal carcinoma; ER, estrogen receptor; PR, progesterone receptor; HER2, human epidermal growth factor receptor 2; SLNB; sentinel lymph node biopsy; RT, radiation therapy; chemo, chemotherapy; MRM, modified radical mastectomy; ALND, axillary lymph node dissection; ROM, range of motion; NED, no evidence of disease.

**Table 2.** Radiation therapy dose-volume results (N = 5).

Characteristic	Patient 1		Patient 2		Patient 3		Patient 4		Patient 5	
Radiation delivery technique	PS	IMRT	PBS	IMRT	PBS	VMAT	PBS	IMRT	PBS	3D CBH
Total dose, GyRBE/fractions	50/25	—	50/25	—	42.4/16	—	50/25	—	50/25	—
Boost	None	—	None	—	Electron scar	—	None	—	None	—
Total boost, Gy/fx	N/A	—	N/A	—	7.95/3	—	N/A	—	N/A	—
Ipsilateral chest wall D95, %	96.0	96.2	97.0	95.7	92.0	94.7	95.4	95.5	95.3	90.5
SCV D95, Gy	48.4	47.4	48.0	48.3	41.5	40.5	46.9	47.7	47.4	45.9
IMN D95, Gy	47.0	47.0	48.7	45.0	41.8	40.3	48.8	47.7	49.6	45.1
Level I-III axilla D95, Gy	48.7	47.7	47.2	47.8	41.6	40.4	47.0	48.2	47.3	47.5
Mean heart dose, Gy	0.0	5.0	1.0	5.0	0.2	2.9	1.0	4.9	1.2	4.2
Heart V5, %	0.0	31.0	4.5	22.5	1.0	15.5	4.5	33.0	5.8	13.8
LAD 0.1-cm^3^ dose, Gy	0.0	28.8	7.5	38.5	8.6	12.2	6.8	29.8	14.2	47.2
Ipsilateral lung V5, %	15.9	26.4	46.0	62.4	55.0^a^	48.7^a^	32.6	62.8	53.4	51.2
Ipsilateral lung V20, %	9.5	66.3	20.0	34.2	31.0^b^	16.6^b^	16.0	22.7	25.7	33.9

**Abbreviations:** PS, passive scattering; IMRT, intensity-modulated radiation therapy; PBS, pencil beam scanning; VMAT, volumetric modulated radiation therapy; 3D, three-dimensional; BH, breath-hold; RBE, relative biological effectiveness; fx, fraction; N/A, not applicable; SCV, supraclavicular node; IMN, internal mammary node; LAD, left anterior descending artery.

Definitions: D95, percentage of total prescription dose to 95% of the volume; V5, volume receiving 5 Gy; V20, volume receiving 20 Gy.

a V4 rather than V5.

b V16 rather than V20.

### Case 1

A 75-year-old White male patient presented with left nipple inversion and a palpable retroareolar mass, with no evidence of nipple discharge. He had a history of insulin-dependent diabetes, obstructive sleep apnea on continuous positive airway pressure, myasthenia gravis on methotrexate, hypertriglyceridemia, and hypertension. His cardiac history included a myocardial infarction 15 years prior, atrial fibrillation with pacemaker placement 3 years prior, and 4 cardiac stents owing to coronary artery disease (the most recent stenting was 3 months before the breast cancer diagnosis). The patient had an extensive family history of breast cancer in females. The patient, who had a 20-pack-year smoking history, quit smoking more than 30 years before.

Breast imaging revealed a 2.1-cm mass in the retroareolar left breast, with left nipple inversion. A left-axillary ultrasound scan was negative for suspicious nodes. An ultrasound-guided biopsy of the palpable left-breast mass yielded a diagnosis of intermediate-grade invasive ductal carcinoma (IDC) that was ER-positive (100%), progesterone receptor (PR)–positive (65%), and HER2neu-negative, with Ki-67 index of 10%. Genetic testing was negative.

This patient underwent a left mastectomy with sentinel lymph node biopsy (SLNB). Pathology revealed American Joint Committee on Cancer (AJCC) 7th edition [13] stage IIB pT2 N1a(sn) cM0 disease, with a 2.5-cm intermediate-grade IDC with lymphovascular space invasion and negative margins, with the closest margin 7 mm posteriorly. One of 4 lymph nodes was positive, containing 0.9 cm of disease with focal extracapsular extension measuring less than 0.1 cm.

Adjuvant antiendocrine therapy was recommended but not chemotherapy. Adjuvant radiation therapy was recommended. The patient’s pacemaker was transferred to the right chest wall before the start of radiation therapy, and the methotrexate was held during radiation therapy.

The left chest wall and comprehensive regional lymphatics were treated to 50 GyRBE at 2 GyRBE per fraction in 25 daily fractions delivered with passive-scattering proton therapy (3 matched fields, with alternating match lines daily). The patient tolerated the radiation therapy well, experiencing acute brisk dermatitis without desquamation (grade 2) and mild esophagitis (grade 1).

With 6.5 years of follow-up, the patient is doing well with no disease recurrence. Cardiac stents were exchanged 3.5 years after radiation therapy. The only late radiation toxicity was grade 1 telangiectasia (**Figure 1**). He completed 5 years of adjuvant letrozole therapy.

### Case 2

A 60-year-old Black male patient presented with a retroareolar left-breast mass with nipple inversion and hyperpigmentation of the surrounding skin. He had a medical history of myocardial infarction, bacterial meningitis, depression, DVT, hyperlipidemia, and hypertension. Contributory family history included a father with a history of unspecified cancer and a mother with a history of stroke. The patient never smoked.

Diagnostic bilateral breast imaging was performed. On the left, the mammogram identified a 1.7 × 2.5–cm mass, confirmed by ultrasonography, which demonstrated a 2.8 × 1.7 × 1.8–cm solid mass in the retroareolar, 2-o’clock position. Evaluation of the left axilla with ultrasonography revealed a single normal-appearing lymph node with a large fatty hilum. Mammography of the right breast demonstrated a 4- to 5-mm round mass in the right retroareolar region.

He underwent ultrasound-guided breast biopsies bilaterally. In the retroareolar left breast, intermediate-grade IDC with mucinous features was present that was ER-positive (90%), PR-positive (50%), and HER-2 negative. In the right breast, grade 2 ductal carcinoma in situ (DCIS) that was ER-positive and PR-positive was identified. Genetic testing identified a variant of uncertain significance (VUS) in the *APC* gene.

The patient underwent a bilateral mastectomy and sentinel lymph node evaluation. Pathology from the left-breast mastectomy specimen demonstrated AJCC 8th edition [14] stage IIA, pT2 N1mic(sn) disease consisting of low-grade IDC, mucinous type, with its largest extent to 3 cm. Three sentinel lymph nodes were removed on the left (one was positive for micrometastases). In the right-breast mastectomy specimen, intermediate-grade DCIS was identified with its greatest extent to 0.4 cm, negative margins (closest, 3 cm), and 4 negative sentinel lymph nodes, indicative of AJCC 8th edition [14] stage 0 pTis N0(sn) disease.

The patient received adjuvant chemotherapy with 4 cycles of docetaxel and cyclophosphamide. Adjuvant radiation was recommended to the left chest wall, and comprehensive regional lymphatics and observation were recommended for the right-breast DCIS. He received 50 GyRBE in 25 daily fractions with PBS. Mometasone was applied twice daily to the irradiated area. He developed grade 1 acute dermatitis.

At 2 years follow-up, the patient remains without evidence of recurrent disease. He continues to take tamoxifen and is active in work and exercise.

### Case 3

The patient was an 80-year-old White male who presented with a painful enlarging left-breast lump and nipple retraction. He had no significant cardiac history, but his family history included a mother who died of a stroke. The patient never smoked, and his genetic testing was negative.

A bilateral diagnostic mammogram and left-breast ultrasonogram demonstrated a retroareolar 2.7 × 1.6–cm mass in the left breast at 3 o’clock, 2 to 3 cm from the nipple. At least 1 morphologically abnormal lymph node was present in the left axilla.

Biopsy of both the left-breast mass and left axillary node revealed intermediate-grade IDC that was ER-positive (>50%), PR-positive (>50%), and Her2neu-negative, with Ki-67 >20%. He started treatment with neoadjuvant tamoxifen.

A positron emission tomography–computed tomography (PET/CT) scan demonstrated left retroareolar breast cancer with multiple suspicious left axillary nodes and a large subcarinal node. The patient underwent an endobronchial ultrasound–guided transbronchial fine-needle aspiration biopsy of the enlarged subcarinal lymph node. Pathology revealed benign bronchial cells, lymphohistiocytic aggregates, and lymphocytes with no malignant cells present.

Repeated PET/CT scans after 3 months of tamoxifen treatment revealed decreased size and fluorodeoxyglucose uptake of the left retroareolar breast neoplasm with ipsilateral nodal disease. No change occurred in the enlarged hypermetabolic subcarinal lymph node. The patient underwent a left modified radical mastectomy. Pathology revealed intermediate-grade IDC, which was 1.7 cm in its greatest dimension. All surgical margins were negative for invasive carcinoma, with the closest margin measuring 1 mm. Extensive angiolymphatic invasion was present. DCIS, intermediate nuclear grade, and cribriform pattern were also present. All surgical margins were uninvolved by DCIS, with the closest margin measuring 4 mm. Level 1 and 2 left axillary lymph node dissection of 20 lymph nodes yielded 18 nodes positive for metastatic ductal carcinoma (18 of 20). The largest focus of metastasis measured 11 mm in its greatest dimension, and extranodal extension was present. The pathologic stage was ypT1c N3a.

Secondary to advanced age, docetaxel and cyclophosphamide were recommended for 6 cycles. Tamoxifen was held while the patient was undergoing chemotherapy. During his course of systemic treatment, he developed pneumonia and extravasation of the chemotherapy into his left arm, leading to lymphedema of the left upper extremity.

Adjuvant radiation therapy to the left chest wall and regional lymphatics was recommended. To minimize the number of appointments for adjuvant radiation therapy, owing to the ongoing COVID-19 pandemic, the chest wall and comprehensive regional lymphatics were treated to 42.4 GyRBE at 2.65 GyRBE per fraction in 16 daily fractions, using PBS consisting of 2 fields. An additional 7.95 Gy at 2.65 Gy per fraction in 3 daily fractions using 6-MeV en face electrons was prescribed to D_max_ (depth of maximum dose) with 0.5-cm bolus to the mastectomy incision. The subcarinal node was not included in the radiation therapy field.

The patient tolerated the radiation well without unexpected toxicity. He developed the anticipated side effects of radiation dermatitis consisting of mild to moderate erythema (grade 1). He had left upper-extremity lymphedema that preceded the start of radiation therapy, and lymphedema measurements did improve during radiation with physical therapy.

With 2.8 years of follow-up, no in-field disease progression has developed. Surveillance PET/CT scans 2 months after radiation showed an increase in the size of a subcarinal lymph node, with subsequent negative biopsy findings of this node on 2 occasions. The subcarinal node has since stabilized. Surveillance chest imaging has demonstrated waxing and waning pulmonary nodules, and none with continuous growth. He continues to receive maintenance systemic therapy with capecitabine with no definite evidence of active disease.

### Case 4

A 41-year-old Black male patient was evaluated in the emergency department for gastrointestinal bleeding. On physical examination, a nurse noted a left-breast mass and nipple retraction. His comorbidities included obesity, hypertension, hypercholesterolemia, and obstructive sleep apnea.

Breast imaging revealed a 2.8 × 3.5 × 1–cm retroareolar left-breast mass. Ultrasonography of the left axilla demonstrated a morphologically normal fat-containing axillary lymph node. A left-nipple punch biopsy revealed Paget disease of the breast and intermediate- to high-grade DCIS arising within a papillary lesion. An ultrasound-guided biopsy of the left-breast mass revealed grade 3 IDC, ER-positive (>90%), PR-negative, Her2neu-negative, with Ki-67 > 20%. Genetic testing was positive for *PALB2* with a VUS in *CHD1*.

He underwent a left mastectomy, SLNB, and a right prophylactic mastectomy. Pathology revealed pT2 N0(sn) disease, with poorly differentiated IDC in the left breast with micropapillary features, measuring 3.7 × 1.8 × 1.4 cm. There was no lymphovascular space invasion. High-grade DCIS and Paget disease of the breast were present, and the margins were negative. Two sentinel nodes were removed, with both testing negative. The right breast was negative for malignancy.

The OncotypeDX@ (Madison, WI) score was 24, and chemotherapy was not recommended. The patient is receiving adjuvant letrozole. Owing to his high-risk, node-negative disease, the patient underwent left chest wall and regional nodal irradiation using PBS to 50 GyRBE in 25 daily fractions. Acute toxicity consisted of grade 2 dermatitis.

Ten months after radiation, the patient’s left chest wall remains mildly hyperpigmented, and he has no evidence of disease progression.

### Case 5

A 73-year-old Black male patient presented with a palpable left retroareolar breast mass (confirmed by a mammogram) that measured 0.9 cm. He underwent an excisional biopsy, followed by a left mastectomy and SLNB. Pathology revealed pT1b NX disease, with grade 3 IDC that was ER-positive (89%-90%), PR-negative, and Her2neu-negative. The primary tumor measured 0.6 cm at its greatest dimension and margins were negative (>2 cm). No lymph nodes were identified in the axillary specimen. Adjuvant therapy consisted of tamoxifen. Genetic testing demonstrated a VUS in the *MSH2* gene.

Two years prior, the patient underwent excision of a low-grade carcinoid tumor of the left lower lobe of the lung. Three months after surgery for breast cancer, he underwent stereotactic body radiotherapy for non–small cell lung cancer in the right upper lobe. That same year, he experienced a myocardial infarction with subsequently reduced ejection fraction (45%-49%). He also had hypertension.

On surveillance imaging for lung cancer 1 year later (compliant with tamoxifen), we identified a 1.5-cm left axillary node as abnormal. A left-axillary ultrasonography was performed, followed by a biopsy of the node. Pathology revealed moderate to poorly differentiated carcinoma, most consistent with a breast primary; this carcinoma was ER-positive (95%), PR-negative, and Her2neu-negative. After systemic imaging with PET confirmed no local recurrence or distant metastatic disease, he underwent left axillary lymph node dissection. Twelve nodes were removed, with 1 containing a tumor measuring 1.5 cm with an extracapsular extension. He developed left upper-extremity lymphedema postoperatively. He then received chemotherapy with 4 cycles of cyclophosphamide and docetaxel.

Left chest wall and regional nodal irradiation were recommended for the patient. Computed tomography simulation was performed with free breathing and breath-hold. Multiple photon techniques were evaluated, and 3D conformal radiation therapy with breath-hold yielded the better of these plans; the mean heart dose measured 4.2 Gy, the 0.1-cm^3^ left anterior descending artery (LAD) dose was 47.4 Gy, and the left lung V20 was 33.9%. Insurance coverage for proton therapy was sought, given his history of 2 prior lung cancers and significant cardiac disease. He started radiation with the photon plan to avoid a delay in care. Insurance coverage was later approved for proton therapy; the mean heart dose was decreased to 1.2 Gy, the 0.1-cm^3^ LAD dose to 14.2 Gy, and the left lung V20 to 25.7% with the proton plan. After 9 fractions, he transitioned to a PBS plan (without breath-hold) for the remaining 16 fractions, resulting in a total dose of 50 Gy in 25 daily fractions. This yielded a composite mean heart dose of 2.3 Gy and a 0.1-cm^3^ LAD dose of 29.4 Gy. He tolerated the radiation well, with toxicity consisting of moderate hyperpigmentation within the radiation field. He is receiving adjuvant therapy with letrozole and leuprorelin.

With 1.5 years of follow-up, the patient remains with no evidence of recurrent breast or lung cancer. He has mild chest wall discomfort, decreased range of motion, and lymphedema of the left upper extremity.

## Discussion

This case series reports the use of proton therapy in treating men with breast cancer. To date, only 1 other case report of a single male patient who had received radiation for breast cancer was identified, which was a case of reirradiation as well [15]. Data on cardiac-sparing radiation therapy techniques in males are minimal, including photon-based cardiac-sparing techniques such as breath-hold, gating, and intensity-modulated radiation therapy. The techniques used for females are extrapolated for use in males. This case series demonstrates the ability of proton therapy to minimize cardiac exposure in males, as has been demonstrated for women in multiple series. In this case series, proton therapy achieved significant cardiac sparing, with a mean heart dose ≤1 GyRBE and a 0.1-cm^3^ LAD dose <15 GyRBE in all patients. This cardiac sparing was achieved without compromising target coverage, including the internal mammary node chain. For 1 very thin patient (case 3), the lung dose increased with the proton plan, but the heart dose was reduced to near zero (0.2 Gy). All other patients benefited from a decreased lung dose in addition to the cardiac sparing with the proton plan.

Darby et al [16] demonstrated that increasing cardiac dose (mean heart dose) was associated with an increased risk of major cardiovascular events, and van den Bogaard et al [17] reported a similar relationship between cardiac radiation exposure and cardiac toxicity. Zureick et al [18] found that LAD D_mean_ EQD_2_ (equivalent dose in 2 Gy fractions) >2.8 Gy, LAD D_max_ EQD_2_ >6.7 Gy, and heart D_mean_ EQD_2_ >0.8 Gy were associated with increased risk of a cardiac event. Proton therapy has been used to decrease the mean heart dose as well as the dose to cardiac substructures, with multiple studies in females demonstrating superior cardiac sparing when compared to photon-based techniques [19–22]. Mean heart doses were reported in a systematic review between 2003 and 2013 by Taylor et al [9], and consistently, proton therapy for regional nodal irradiation yielded lower mean heart doses than photon techniques in patients with breast cancer.

Existing data suggest a benefit to adjuvant radiation therapy for men, similar to findings noted in women. Yadav et al [7] reported that receipt of radiation therapy, as well as chemotherapy and endocrine therapy, was independently associated with increased overall survival rates for men with breast cancer. Yadav et al [7] reported an increase in radiation therapy use after both breast-conserving surgery and mastectomy in male patients with breast cancer, with a strong survival benefit identified in the cohort undergoing radiation therapy after breast-conserving surgery. Nevertheless, radiation therapy may be underused in men with breast cancer. One international series of more than 1400 men with breast cancer found that 45% of men treated with lumpectomy and 31% with node-positive disease treated with mastectomy did not undergo adjuvant radiation therapy [23]. In a Surveillance, Epidemiology, and End Results analysis assessing men and women with breast cancer diagnosed between 2005 and 2010, there was a 5% difference in survival rates between men and women (82.8% vs 88.5%), with a 43% greater risk of death in males than females during the follow-up period that extended through 2015 [24]. Her2-positive and triple-negative disease are variables associated with inferior survival rates in men with breast cancer [25], similar to women with breast cancer.

## Conclusion

In a small case series for a rare diagnosis in men, proton therapy to the chest wall and regional lymphatics, including internal mammary nodes, resulted in low cardiac exposure, high local regional disease control rates, and minimal toxicity. This case series supports the use of proton therapy in treating males with breast cancer undergoing regional nodal irradiation to achieve maximal cardiac sparing.

## References

[1] Anderson WF Althuis MD Brinton LA Devesa SS Is male breast cancer similar or different than female breast cancer? *Breast Cancer Res Treat* 2004 83 77–86 14997057 10.1023/B:BREA.0000010701.08825.2d

[2] Wu P He D Zhu S Chang H Wang Q Shao Q Li G The role of postoperative radiation therapy in stage I-III male breast cancer: a population-based study from the Surveillance, Epidemiology, and End Results database *Breast* 2022 65 41–8 35810531 10.1016/j.breast.2022.06.004 PMC9272391

[3] Siegel RL Miller KD Fuchs HE Jemal A Cancer statistics, 2021 *CA Cancer J Clin* 2021 71 7–33 33433946 10.3322/caac.21654

[4] Speirs V Shaaban AM The rising incidence of male breast cancer *Breast Cancer Res Treat* 2009 115 429–30 18478326 10.1007/s10549-008-0053-y

[5] Abdelwahab Yousef AJ Male breast cancer: epidemiology and risk factors *Semin Oncol* 2017 44 267–72 29526255 10.1053/j.seminoncol.2017.11.002

[6] Yalaza M Inan A Bozer M Male breast cancer *J Breast Health* 2016 12 1–8 28331724 10.5152/tjbh.2015.2711 PMC5351429

[7] Yadav S Karam D Bin Riaz I Xie H Durani U Duma N Giridhar KV Hieken TJ Boughey JC Mutter RW Hawse JR Jimenez RE Couch FJ Leon-Ferre RA Ruddy KJ Male breast cancer in the United States: treatment patterns and prognostic factors in the 21st century *Cancer* 2020 126 26–36 31588557 10.1002/cncr.32472 PMC7668385

[8] Gucalp A Traina TA Eisner JR Parker JS Selitsky SR Park BH Elias AD Baskin-Bey ES Cardoso F Male breast cancer: a disease distinct from female breast cancer *Breast Cancer Res Treat* 2019 173 37–48 30267249 10.1007/s10549-018-4921-9 PMC7513797

[9] Taylor CW Wang Z Macaulay E Jagsi R Duane F Darby SC Exposure of the heart in breast cancer radiation therapy: a systematic review of heart doses published during 2003 to 2013 *Int J Radiat Oncol Biol Phys* 2015 93 845–53 26530753 10.1016/j.ijrobp.2015.07.2292

[10] Centers for Disease Control and Prevention (CDC) Men and heart disease October 14, 2022. Accessed March 1, 2023. https://www.cdc.gov/heartdisease/men.htm

[11] Curtin SC Trends in cancer and heart disease death rates among adults aged 45-64: United States, 1999-2017 *Natl Vital Stat Rep* 2019 68 1–9 32501204

[12] NRG Oncology Breast Contouring RADCOM Consortium v3 February 23, 2016. https://imsva91-ctp.trendmicro.com:443/wis/clicktime/v1/query?url=https%3a%2f%2fwww.nrgoncology.org%2fPortals%2f0%2fScientific%2520Program%2fCIRO%2fAtlases%2fRADCOMP%2fRADCOMP%2520Breast%2520Atlas%2520v.3%2520%2d%2520bigreduced.pdf%3fver%3d2020%2d08%2d01%2d140849%2d360&umid=68A0644F-05E5-5806-86B8-129E0C502999&auth=5ab06289d9c3b14f9a77f69d29e7a25870e86301-bb68c94ce9b005d551cc0ab6f2f36b7fe0c70d47. Accessed April 1, 2023

[13] Edge SB Byrd DR Compton CC Fritz AG Greene FL Trotti A , editors. *AJCC cancer staging manual* ( 7th ed). New York, NY Springer 2010.

[14] Amin MB Edge S Greene F Byrd DR Brookland RK Washington MK Gershenwald JE Compton CC Hess KR et al. (Eds.). *AJCC Cancer Staging Manual* ( 8th edition). Springer International Publishing: American Joint Commission on Cancer 2017.

[15] Giap BQ Giap F Einck JP LePage R Blasongame DM Waldinger A Dong L Mascia A Chang A Rossi CJ Giap H A Case Study: Proton Therapy for Male Breast Cancer with Previous Irradiation *Int J Part Ther* 2016 2 579–83 31772969 10.14338/IJPT-15-00031.1 PMC6871644

[16] Darby SC Ewertz M McGale P Bennet AM Blom-Goldman U Bronnum D Correa C Cutter D Gagliardi G Gigante B Jensen MB Nisbet A Peto R Rahimi K Taylor C Hall P Risk of ischemic heart disease in women after radiotherapy for breast cancer *N Engl J Med* 2013 368 987–98 23484825 10.1056/NEJMoa1209825

[17] van den Bogaard VA Ta BD van der Schaaf A Bouma AB Middag AM Bantema-Joppe EJ van Dijk LV van Dijk-Peters FB Marteijn LA de Bock GH Burgerhof JG Gietema JA Langendijk JA Maduro JH Crijns AP Validation and Modification of a Prediction Model for Acute Cardiac Events in Patients With Breast Cancer Treated With Radiotherapy Based on Three-Dimensional Dose Distributions to Cardiac Substructures *J Clin Oncol* 2017 35 1171–8 28095159 10.1200/JCO.2016.69.8480 PMC5455600

[18] Zureick AH Grzywacz VP Almahariq MF Silverman BR Vayntraub A Chen PY Gustafson GS Jawad MS Dilworth JT Dose to the Left Anterior Descending Artery Correlates With Cardiac Events After Irradiation for Breast Cancer *Int J Radiat Oncol Biol Phys* 2022 114 130–9 35483540 10.1016/j.ijrobp.2022.04.019

[19] Lin LL Vennarini S Dimofte A Ravanelli D Shillington K Batra S Tochner Z Both S Freedman G Proton beam versus photon beam dose to the heart and left anterior descending artery for left-sided breast cancer *Acta Oncol* 2015 54 1032–9 25789715 10.3109/0284186X.2015.1011756

[20] Lee HL Lim LH Master Z Wong SMM The role of breath hold intensity modulated proton therapy for a case of left-sided breast cancer with IMN involvement. How protons compare with other conformal techniques? *Tech Innov Patient Support Radiat Oncol* 2020 15 1–5 32490219 10.1016/j.tipsro.2020.03.001 PMC7256639

[21] Xu N Ho MW Li Z Morris CG Mendenhall NP Can proton therapy improve the therapeutic ratio in breast cancer patients at risk for nodal disease? *Am J Clin Oncol* 2014 37 568–74 23466577 10.1097/COC.0b013e318280d614

[22] Depauw N Batin E Daartz J Rosenfeld A Adams J Kooy H MacDonald S Lu HM A novel approach to postmastectomy radiation therapy using scanned proton beams *Int J Radiat Oncol Biol Phys* 2015 91 427–34 25636765 10.1016/j.ijrobp.2014.10.039

[23] Cardoso F Bartlett JMS Slaets L van Deurzen CHM van Leeuwen-Stok E Porter P Linderholm B Hedenfalk I Schroder C Martens J Bayani J van Asperen C Murray M Hudis C Middleton L Vermeij J Punie K Fraser J Nowaczyk M Rubio IT Aebi S Kelly C Ruddy KJ Winer E Nilsson C Lago LD Korde L Benstead K Bogler O Goulioti T Peric A Litiere S Aalders KC Poncet C Tryfonidis K Giordano SH Characterization of male breast cancer: results of the EORTC 10085/TBCRC/BIG/NABCG International Male Breast Cancer Program *Ann Oncol* 2018 29 405–17 29092024 10.1093/annonc/mdx651 PMC5834077

[24] Liu N Johnson KJ Ma CX Male breast cancer: an updated Surveillance, Epidemiology, and End Results data analysis *Clin Breast Cancer* 2018 18 e997–1002 30007834 10.1016/j.clbc.2018.06.013

[25] Leone J Freedman RA Lin NU Tolaney SM Vallejo CT Leone BA Winer EP Leone JP Tumor subtypes and survival in male breast cancer *Breast Cancer Res Treat* 2021 188 695–702 33770314 10.1007/s10549-021-06182-y

